# Jumping to Conclusions: Mechanisms of Cognitive Control in Decision-Making Under Uncertainty

**DOI:** 10.3390/bs15020226

**Published:** 2025-02-17

**Authors:** Pei-Chun Shih, África Pérez-Santiago, Daniel Peña, Daniel Wazne, Santiago Román

**Affiliations:** 1 Department of Biological and Health Psychology, Universidad Autonoma de Madrid, 28049 Madrid, Spain; aperez@fudepi.org (Á.P.-S.); dwazne@apertia-consulting.com (D.W.); 2 Fundación Universitaria Para el Desarrollo de la Psicología y la Investigación, 28016 Madrid, Spain; dpena@apertia-consulting.com (D.P.); lsantirogue.arc@gmail.com (S.R.)

**Keywords:** jumping to conclusions, uncertainty, cognitive control, bis/bas, Box Task

## Abstract

Cognitive control encompasses mental processes that regulate thoughts and actions to achieve specific goals. It is essential in decision-making, facilitating attention management, impulse inhibition, and adaptation to new information—skills critical for rational choices, particularly under uncertainty. In jumping to conclusions (JTC), where individuals make premature decisions based on limited evidence, cognitive control deficits are often implicated. Cognitive stability helps maintain focus and resist distractions but may lead to premature conclusions if excessive. In contrast, cognitive flexibility supports adaptive decision-making by incorporating new information but may foster hasty decisions if insufficient. This study examined JTC and cognitive control mechanisms in 200 university students. Participants completed the Box Task under varied uncertainty conditions, tasks assessing stability (Flanker–Simon Task) and flexibility (Letter–Number Task), and the BIS/BAS questionnaire. Cluster analysis identified three evidence-gathering profiles: minimal, moderate, and extensive. Significant differences were found between clusters in both flexibility and stability, with individuals in the extensive evidence-gathering cluster showing superior cognitive control. However, higher uncertainty amplified the reliance on flexibility, highlighting its role in adapting to challenging conditions, while stability remained unaffected. No significant association emerged between BIS/BAS indices and Box Task performance. These findings emphasize the interplay between uncertainty, cognitive control, and decision-making, underscoring the need for further research to refine interventions targeting decision-making biases.

## 1. Introduction

Decision-making is a multifaceted cognitive process that enables individuals to evaluate options and select the most suitable course of action. This process is essential to human behavior as it facilitates reasoning under uncertainty, weighing the desirability and probabilities of different alternatives (De Winkel et al., 2017; Kovács et al., 2017; Li et al., 2019). It becomes particularly crucial in scenarios where no inherently correct choices are available to guide decisions (Jamali et al., 2019). Such situations are ubiquitous in real-world settings, underscoring the importance of aligning experimental paradigms with the complexities of everyday environments to ensure ecological validity (Buelow et al., 2024). This alignment enhances the applicability of research findings to practical domains, including clinical, financial, and organizational settings (Schmuckler, 2001).

For instance, in financial investments, individuals must assess risks with incomplete information (Fenton-O’Creevy et al., 2011), while professionals in high-stakes environments such as air traffic control and emergency medicine must make rapid yet precise decisions to ensure safety (Flin et al., 2008). Additionally, in social and political contexts, reliance on limited evidence can shape public opinion, contributing to the spread of misinformation or belief in conspiracy theories, particularly in times of uncertainty (Bronstein et al., 2019; Pytlik et al., 2020; van Prooijen & Douglas, 2017).

Understanding decision-making processes has profound implications for both theoretical frameworks and practical applications. Investigating how individuals respond to uncertainty and prioritize evidence gathering provides insight into the mechanisms underlying adaptive and maladaptive behaviors. These insights are pivotal for designing interventions aimed at improving decision-making across a wide range of contexts, from clinical settings to everyday decision-making scenarios.

Efforts to explore decision-making have primarily relied on two methodological approaches: self-report questionnaires and cognitive tasks. Self-report measures, such as the Melbourne Decision-Making Questionnaire (Mann et al., 1997) or the General Decision-Making Style scale (Scott & Bruce, 1995), focus on dispositional dimensions of decision-making (Aluja et al., 2024). In contrast, cognitive tasks examine dynamic decision-making under conditions of uncertainty or risk (Brand et al., 2006). Among these, the Iowa Gambling Task (Bechara et al., 2000; Dunn et al., 2006) has been particularly influential in assessing decision-making under ambiguity, requiring participants to learn from feedback to avoid suboptimal choices and prioritize advantageous ones (Cassotti et al., 2014).

While self-report measures have provided insights into dispositional predictors of behaviors such as economic decisions (García-Gallego et al., 2017), health outcomes (Bavoľár & Bačíkova-Slešková, 2018), and problematic smartphone use (Urieta et al., 2022), cognitive tasks are indispensable for uncovering the mechanisms driving these behaviors. Specifically, they help elucidate how individuals focus on relevant information, suppress irrelevant stimuli, and adapt to changing contexts.

Cognitive control, which regulates thought and action in complex environments, is a fundamental construct in decision-making research (Badre, 2025; Kool & Botvinick, 2018; Miller & Cohen, 2001). It consists of two complementary components: cognitive stability and cognitive flexibility (Egner, 2023). Stability maintains persistence in goal-directed actions, while flexibility allows adaptation to new information. These processes rely on working memory, which updates task goals and shifts attention as needed (Cowan, 1988). Proper regulation of cognitive stability and flexibility ensures adaptive behavior, whereas dysregulation may result in excessive rigidity or distractibility, contributing to cognitive impairments in clinical conditions (Uddin, 2021).

Task switching, which relies on cognitive flexibility, is commonly evaluated using behavioral tasks. One such paradigm is the Letter–Number Task (Rogers & Monsell, 1995), which assesses switching ability by requiring participants to shift attention between categorization rules depending on the position of number–letter pairs on the screen. Impaired switching is associated with increased reaction times (RTs) and higher error rates in switch trials compared to non-switch trials (Egner & Siqi-Liu, 2024; Miyake et al., 2000). Additionally, cognitive load and time pressure can amplify these costs, making the task useful for assessing flexibility in decision-making contexts.

The Flanker Task (Eriksen & Eriksen, 1974) is another well-established paradigm for evaluating cognitive control, where participants respond to a central target while ignoring adjacent distractors. A variant incorporating elements of the Simon Task (Simon, 1982) introduces spatial cues influencing response selection (Stoffels & van der Molen, 1988). Typically, incongruent trials lead to longer RTs than congruent ones due to increased cognitive demands. However, in combined Flanker–Simon tasks, this pattern can reverse: in incongruent Flanker trials, RTs may be longer even in congruent Simon conditions. This suggests that the simultaneous processing of spatial and stimulus-related conflicts alters response patterns, likely because the cognitive system prioritizes conflict resolution over simple congruency effects. Task-irrelevant stimulus features (e.g., stimulus location in the Simon Task, flanker identity in the Flanker Task) can either facilitate or interfere with response tendencies (Ridderinkhof et al., 2020). The integration of these paradigms enables a more systematic evaluation of cognitive inhibition and control.

An example of decision-making bias associated with maladaptive outcomes is jumping to conclusions (JTC), a tendency to form premature judgments based on insufficient evidence. JTC has been extensively studied in clinical populations, particularly among individuals with schizophrenia or delusional tendencies (Dudley et al., 2016; Fine et al., 2007; Freeman & Garety, 2014; P. A. Garety et al., 2005; P. Garety et al., 2013; Krężołek et al., 2019). However, it is also observed in non-clinical populations, especially in tasks requiring rapid decisions or those involving deliberately induced ambiguity (Balzan et al., 2017; Klevjer & Pfuhl, 2023). Research suggests that JTC may be associated with deficits in cognitive flexibility, which could hinder the ability to integrate additional evidence and adjust initial judgments when presented with new information (Balzan et al., 2017; Andreou et al., 2015). Additionally, while the role of excessive cognitive stability in JTC remains less established, it has been hypothesized that a rigid adherence to initial impressions may limit the capacity for reevaluation, particularly in contexts requiring dynamic adjustments to new evidence. Neuroimaging findings indicate that disruptions in cognitive control networks, including the prefrontal cortex and anterior cingulate cortex, are associated with JTC behaviors (Andreou et al., 2018; Furl & Averbeck, 2011; Ridderinkhof et al., 2004). These findings underscore the importance of investigating specific cognitive control mechanisms in the context of JTC, as well as how these processes may be influenced by external demands and individual differences.

To study the jumping-to-conclusions (JTC) bias, researchers have relied on probabilistic reasoning tasks to achieve objective assessment. The most widely recognized and utilized is the Beads Task (Huq et al., 1988), in which participants are presented with two jars, each containing two types of stimuli (colored beads) in distinct ratios. The jars remain concealed from view, and beads are sequentially drawn from one of them in a seemingly random yet predetermined order. Following each draw, participants are prompted to indicate whether they have identified the jar from which the beads are being selected. The JTC bias is reflected in participants who require fewer draws to reach a decision, often making judgments based on limited evidence (McLean et al., 2017).

Alternatively, an adaptation of the Beads Task, known as the Box Task, has been proposed as conceptually simpler and experimentally more versatile (Balzan et al., 2017). In the Box Task, participants are shown a grid of concealed boxes, each hiding one of two colors, with one color always in the majority. Unlike the Beads Task, which follows a predetermined sequence, the Box Task allows participants to uncover boxes at their own pace before determining which color predominates. This feature ensures that the total amount of evidence is available from the outset, reinforcing the idea that participants have the opportunity to consider all available information. Consequently, a participant who reaches a decision after uncovering fewer boxes exemplifies a more direct expression of the JTC bias in decision-making (Balzan et al., 2017). These modifications to the original task design enable the systematic manipulation of task parameters in the Box Task, such as levels of uncertainty and difficulty, making it a valuable tool for examining decision-making processes under different cognitive demands.

The relevance of the Box Task for studying JTC bias has been further supported by neuroimaging research. Andreou et al. (2018) demonstrated that decision-making data obtained using the Box Task were associated with the functional connectivity of task-positive and task-negative networks. This finding highlights its potential for investigating the neural mechanisms underlying hasty decision-making. Given its adaptability and capacity to capture individual differences in evidence evaluation, the Box Task offers a promising framework for exploring the role of cognitive control in mitigating premature decisions and fostering more adaptive decision-making behaviors.

Personality traits influence decision-making tendencies by shaping cognitive control processes. Anxiety, often conceptualized as a dispositional trait, can impair cognitive control by increasing sensitivity to potential threats and punishment, leading to excessive caution and hesitation. Conversely, impulsivity undermines self-regulation by prioritizing immediate rewards over long-term goals (Starcke & Brand, 2012). Gray’s Behavioral Inhibition System (BIS) and Behavioral Approach System (BAS) provide a theoretical framework for understanding these influences (Corr, 2004; Gray, 1990).

Anxiety is strongly associated with attentional biases towards negative stimuli, fostering avoidance behaviors and impairing decision-making efficiency (Eysenck et al., 2007; Eysenck & Derakshan, 2011; Mathews & MacLeod, 2005). BIS, characterized by sensitivity to punishment and risk aversion, is closely linked to anxious responses, promoting vigilance and cognitive stability as a means of mitigating perceived threats. However, these adaptive features often come at the expense of flexibility, as high trait anxiety can make it difficult to ignore threat-related cues, reducing adaptability in uncertain environments (Bijttebier et al., 2009).

In contrast, BAS, associated with reward sensitivity and goal-directed exploration, enhances cognitive flexibility and bold decision-making but can also increase impulsivity and premature actions when reward sensitivity outweighs evaluative processes (Carver & White, 1994; Corr, 2004). This effect is particularly pronounced in tasks involving immediate gratification, where impulsivity results in insufficient consideration of long-term outcomes (Heym et al., 2008; Smillie et al., 2006). Moreover, BAS-driven impulsivity is linked to reduced inhibitory control, contrasting with the overregulation observed in BIS-dominated anxiety (Perkins et al., 2007).

Given these theoretical foundations, this study aims to explore the interplay between JTC, cognitive control, and BIS/BAS traits. Specifically, it examines how cognitive stability and flexibility, assessed through the Flanker–Simon and Letter–Number Tasks, interact with personality traits to influence JTC tendencies in the Box Task. By integrating cognitive and personality frameworks, this research seeks to refine our understanding of JTC mechanisms and their implications for decision-making under uncertainty. Additionally, it addresses critical gaps in the literature by investigating the moderating role of BIS/BAS traits in the relationship between cognitive control and JTC. This approach provides a comprehensive perspective on individual differences in decision-making and contributes to identifying the cognitive and personality factors that underlie maladaptive decision-making patterns.

## 2. Materials and Methods

### 2.1. Participants

The sample consisted of 200 first-year psychology students recruited from a university setting. To ensure task engagement and data quality, exclusion criteria were established for each task based on specific performance thresholds. These criteria were designed to identify cases where task execution was inadequate, such as high error rates or inconsistent responses, and will be detailed in the Instruments and Measures section. Following the application of these criteria, the final sample size varied slightly across tasks depending on the availability of valid data. The sample was predominantly female (82.5%), with a mean age of 18.78 years (SD = 3.53).

### 2.2. Instruments and Measurements

The study employed well-established tasks and instruments, supported by existing literature, to assess psychological and behavioral constructs.

#### 2.2.1. BIS-BAS Questionnaire

The BIS-BAS Questionnaire (Carver & White, 1994; Spanish version by Torrubia et al., 2001) assesses individual differences in personality traits related to sensitivity to punishment and reward. It consists of 20 items divided into two main scales: (1) the Behavioral Inhibition System (BIS), which evaluates sensitivity to punishment and the propensity for cautious, avoidance-oriented behavior, and (2) the Behavioral Activation System (BAS), which measures sensitivity to reward and different dimensions of goal-directed, approach-oriented behavior.

#### 2.2.2. Box Task

**Figure 1:**
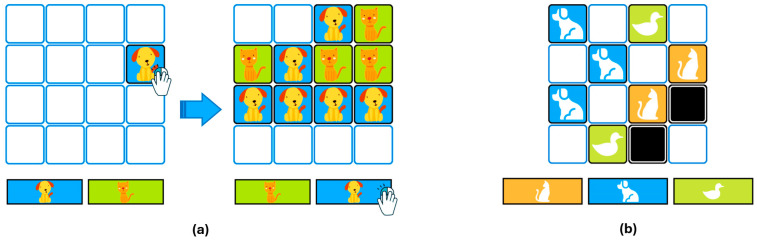
Illustration of the Box Task: (**a**) Condition 1 with fully informative boxes; (**b**) Condition 2 with partially uninformative boxes.

The Box Task, adapted from Balzan et al. (2017), was designed to evaluate decision-making under uncertainty and evidence-gathering behavior, specifically the tendency to jump to conclusions. In this task, participants were required to decide which category—dogs, cats, or ducks—was the majority. Participants gathered information by clicking on boxes displayed on a grid, revealing their contents (a dog, a cat, or a duck). Participants could continue opening boxes until they felt confident enough to make a decision (see Figure 1).

The task consisted of three experimental conditions, each presented in six trials, progressively increasing in difficulty based on the number of decision options, the number of boxes in the grid, and the level of uncertainty introduced.

In Condition 1, participants decided whether there were more dogs or cats. The grid contained 16 boxes, each of which revealed its content when clicked (see Figure 1a). In this condition, every box provided information, making it the simplest and least ambiguous condition.

In Condition 2, participants decided whether there were more dogs, cats, or ducks. The grid consisted of 16 boxes, but with an added layer of uncertainty. In each trial, a specific number of boxes failed to reveal any information when opened (see Figure 1b). Specifically, in three trials, 4 boxes provided no information, while in the remaining three trials, this number increased to 6. This additional uncertainty made it more challenging to determine the majority category compared to Condition 1.

In Condition 3, participants again decided whether there were more dogs, cats, or ducks. However, the grid size increased to 25 boxes, requiring participants to process more evidence. Similar to Condition 2, this condition included trials with differing numbers of uninformative boxes: 6 in some trials and 9 in others. These features further heightened uncertainty and cognitive load. Condition 3 was the most complex, combining a larger grid with an increased number of uninformative boxes, making it more difficult to synthesize information and identify the majority category.

Following the guidelines proposed by Cattell on the appropriate use of objective tests (Cattell & Warburton, 1967), feedback is intentionally withheld during the execution of the Box Task to ensure that the observed decision-making processes reflect intrinsic behavioral patterns without being influenced by external cues. The sequence of stimuli was identical for all participants, regardless of the order of boxes selected, to guarantee a consistent dynamic interaction with the task across all subjects.

The number of boxes opened, recorded in each condition, served as a measure of jumping to conclusions, with fewer boxes indicating a stronger tendency. Participants who failed to open any boxes in a trial were excluded from the analysis for that condition.

#### 2.2.3. Letter–Number Task

In the Letter–Number task, adapted from Miyake et al. (2000), participants were presented with a letter–number pair (e.g., 7G) in one of four quadrants on a computer screen. The instructions varied based on the quadrant in which the pair appeared: when the pair was presented in either of the top two quadrants, participants were required to indicate whether the number was odd or even (2, 4, 6, and 8 for even; 3, 5, 7, and 9 for odd). Conversely, when the pair appeared in either of the bottom two quadrants, participants were instructed to determine whether the letter was a consonant or a vowel (G, K, M, and R for consonants; A, E, I, and U for vowels).

The task consisted of two blocks: a training block and a main block. The training block included 32 trials divided into three phases. In the first phase (8 trials), letter–number pairs were presented exclusively in the top two quadrants, requiring participants to categorize numbers without task switching. In the second phase (8 trials), the pairs were displayed only in the bottom two quadrants, requiring participants to categorize letters. In the third phase (16 trials), the pairs were randomly presented across all four quadrants, introducing the need to alternate between number and letter categorization depending on their location.

The main block comprised 64 trials, with letter–number pairs appearing in a randomized order across all four quadrants. Half of the trials required participants to mentally switch between categorizing numbers (top quadrants) and letters (bottom quadrants), while the other half did not involve a task switch.

The primary measures for this task were mental shift cost and task accuracy. Mental shift cost was calculated as the difference in average reaction times (RTs) between trials that required a mental switch (switching between number and letter categorization) and those that did not. Accuracy was determined by the percentage of correct responses across all trials. Participants with an error rate exceeding 15% were excluded from the analysis to ensure data reliability.

#### 2.2.4. Flanker–Simon Task

**Figure 2:**
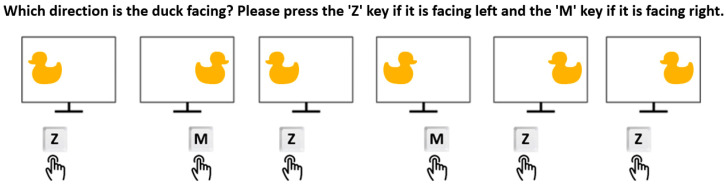
Flanker–Simon Task stimuli and response mapping for duck’s beak direction.

The Flanker–Simon Task, adapted for this study, is based on the classical paradigm introduced by Eriksen and Eriksen (1974) and evaluates attentional control and conflict resolution. In this version, participants were presented with a duck image on the screen, with its beak pointing either to the left or the right. The task required participants to indicate the direction of the duck’s beak by pressing the “Z” key for left and the “M” key for right, regardless of the duck’s position on the screen (left or right). The challenge lay in ignoring the positional congruence or incongruence between the duck’s location and the direction of its beak, focusing solely on the beak’s orientation. This design is conceptually similar to the Simon Task (Simon, 1982), where spatial and directional conflicts are central to assessing cognitive control (see Figure 2).

The task consisted of 140 trials divided into training and testing phases. During the 20 trials of the training phase, participants familiarized themselves with the task requirements, receiving feedback to ensure clarity and accuracy. In the testing phase, comprising 90 trials, stimuli were presented in a randomized order, with half being congruent (the duck’s position matched the direction of its beak) and half incongruent (the duck’s position did not match the direction of its beak). In total, 30 additional stop-signal trials were embedded within the testing phase.

The primary measures for this task were accuracy and the congruency effect. Accuracy was calculated as the proportion of correct responses during the testing phase, serving as an indicator of attentional control. The congruency effect, reflecting the additional cognitive effort required to resolve conflicts between positional and directional information, was measured as the difference in reaction times (RTs) between congruent and incongruent trials. Participants with an error rate exceeding 15% during the testing phase were excluded from the analysis to ensure data reliability.

### 2.3. Procedure

All tasks were administered online using the LimeSurvey platform in two sessions separated by one week. In the first session, participants completed the Flanker–Simon Task, the Box Task, and the BIS-BAS Questionnaire. The second session included the Letter–Number Task, along with other unrelated tasks. Students completed these activities as part of their practical coursework, but only those who provided informed consent to participate in the research were included in the study.

To ensure consistency, participants were instructed to complete the tasks in a quiet environment free from distractions, using a desktop or laptop computer. They were given detailed instructions for each task at the beginning of the session, and practice trials were included where applicable to familiarize them with the procedures. The order of tasks within each session was fixed to minimize variability across participants. Completion times for each session ranged between 30 and 45 min.

### 2.4. Data Analysis

Data were analyzed using SPSS (Version 28). Descriptive statistics and Pearson correlations were initially computed to summarize overall performance and examine relationships between performance in the Box Task and other variables, including personality traits and cognitive control measures. These exploratory analyses provided a foundational understanding of the data and the connections between variables.

A hierarchical cluster analysis was performed to identify homogeneous patterns of exploratory behavior in the Box Task across the three experimental conditions. Using Ward’s method with squared Euclidean distance, clusters were formed, with the optimal number determined by examining dendrograms and agglomeration coefficients. This step allowed for the classification of participants into distinct groups based on their performance metrics in the Box Task. To further examine differences between these clusters, one-way ANOVA tests were conducted on key variables to determine significant distinctions across the identified groups.

Following the cluster analysis, a multinomial logistic regression was conducted to evaluate the predictive value of relevant covariates for cluster membership in the Box Task. Only variables that showed significant differences between the identified clusters were included as predictors in the regression model. This ensured that the analysis focused on meaningful distinctions across groups and enhanced the explanatory power of the results. By integrating these steps, the analysis aimed to uncover the relationships between cognitive processes, personality factors, and exploratory behavior in decision-making.

## 3. Results

Table 1 presents the descriptive statistics for the primary variables across the three conditions of the Box Task. As task complexity increased, the number of boxes opened rose progressively, reflecting greater evidence-gathering behavior in response to heightened demands. However, the distribution of this variable shifted notably across conditions. While the distribution in Condition 1 was approximately normal, with a broader spread of cases observed across the range of values for the variable, Conditions 2 and 3 exhibited increasing skewness and kurtosis, forming a J-shaped pattern with a concentration of participants opening most or all available boxes. This shift suggests that under conditions of greater uncertainty, participants tended to adopt a cautious strategy, prioritizing extensive evidence gathering before making a decision.

Strong and statistically significant correlations between the number of boxes opened across conditions further indicate consistency in individual evidence-gathering strategies, despite variations in task demands. These patterns highlight stable individual differences in decision-making behavior, as well as adaptive adjustments to increasing levels of uncertainty. Notably, some participants maintained a balanced approach, while others maximized evidence collection to prioritize certainty or opened the fewest boxes, indicative of a strong “jumping to conclusions” tendency.

These observed patterns in evidence-gathering behavior were further clarified through a hierarchical cluster analysis using Ward’s method. The optimal number of clusters was determined based on the agglomeration coefficients, with a marked increase observed when transitioning from three to two clusters (coefficient = 601.348), supporting the selection of a three-cluster solution. To further evaluate the clustering results, inter-cluster distances were calculated to assess the degree of separation between clusters. The distances ranged from 7.31 to 15.94, indicating a meaningful level of differentiation. Cluster 1 and Cluster 2 were the closest (distance = 7.31), whereas Cluster 1 and Cluster 3 exhibited the greatest separation (distance = 15.94), underscoring the distinctiveness of Cluster 3 within the variable space.

**Figure 3:**
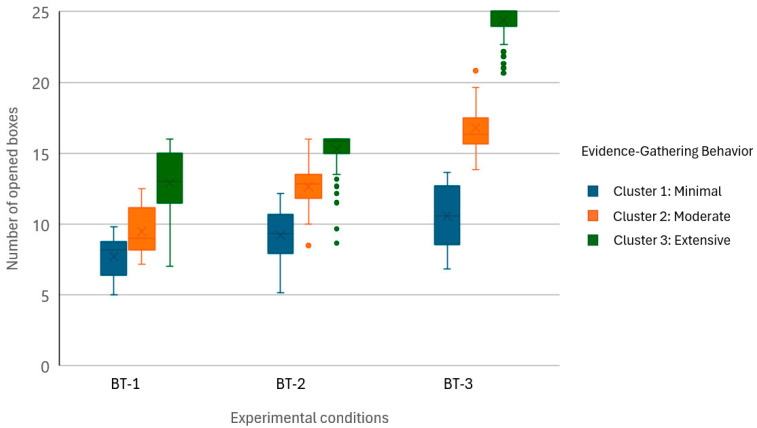
Box plot of evidence-gathering behavior across clusters in the three Box Task conditions. The whiskers in the box plots represent the range of values within 1.5 times the interquartile range (IQR). Outliers are displayed as individual points outside this range.

Table 2 summarizes the characteristics of the three clusters identified through the analysis (see Figure 3). The Minimal Evidence Gatherers (Cluster 1) opened the fewest boxes across all conditions, reflecting a strong tendency toward jumping to conclusions. The Moderate Evidence Gatherers (Cluster 2) adopted a more balanced evidence-gathering strategy, achieving a trade-off between task performance and efficiency. The Extensive Evidence Gatherers (Cluster 3) opened the most boxes, reflecting a cautious and comprehensive strategy aimed at minimizing uncertainty. Significant differences were observed in the number of boxes opened across clusters (*p* < 0.001) and in measures of cognitive flexibility (Letter–Number Task) and cognitive stability (Flanker–Simon Task), with the Extensive Evidence Gatherers showing superior performance across these domains.

It should be emphasized that there was a notable disparity in the size of the clusters. The Minimal Evidence Gatherers included 22 participants (11.4%), the Moderate Evidence Gatherers comprised 23 participants (11.9%), and the Extensive Evidence Gatherers were the largest, with 138 participants (71.5%). This imbalance likely reflects the greater prevalence of evidence-gathering strategies consistent with the Extensive Evidence Gatherers, suggesting that a cautious approach to decision-making is more common in the sample. However, the smaller sizes of the Minimal and Moderate Evidence Gatherers underline the importance of considering individual differences and less frequent decision-making strategies.

To examine the associations between cognitive control mechanisms and cluster memberships, a multinomial logistic regression analysis was conducted. The model was statistically significant (−2*LL* = 207.634, *χ*^2^ = 24.679, *df* = 6, *p* < 0.001), with pseudo-*R*^2^ values indicating acceptable explained variance (Cox and Snell *R*^2^ = 0.142, Nagelkerke *R*^2^ = 0.186, McFadden *R*^2^ = 0.106). Table 3 provides a comprehensive summary of the predictors across all cluster comparisons. Correct responses in the Flanker–Simon Task significantly reduced the odds of belonging to Cluster 2 (Moderate) compared to Cluster 1 (Minimal) by 21.3% (OR = 0.787, *p* = 0.042). In contrast, the difference in response latency between non-switching and switching trials in the Letter–Number Task positively predicted membership in Cluster 2 (Moderate), with each millisecond increase in latency raising the odds by 0.4% (OR = 1.004, *p* = 0.006). For Cluster 3 (Extensive), correct responses in the Letter–Number Task increased the odds of membership compared to Cluster 1 by 30.2% (OR = 1.302, *p* = 0.026), while response latency differences also contributed positively, increasing the odds by 0.3% per millisecond (OR = 1.003, *p* = 0.006). For the comparison between Cluster 3 (Extensive) and Cluster 2 (Moderate), correct responses in the Letter–Number Task were negatively associated with membership in Cluster 3, decreasing the odds by 22.6% (OR = 0.774, *p* = 0.017). No significant predictors were observed in the Flanker–Simon Task for the comparison between Cluster 3 and Cluster 2. These results highlight the role of cognitive stability (Flanker–Simon Task) and flexibility (Letter–Number Task) in differentiating decision-making strategies under uncertainty, with distinct patterns emerging across clusters. Specifically, the Extensive Evidence Gatherers (Cluster 3) exhibited a cautious and comprehensive approach, opening the most boxes in the Box Task and showing superior cognitive flexibility, as indicated by their higher performance in the Letter–Number Task, alongside relatively lower cognitive stability, as reflected in their performance on the Flanker–Simon Task.

## 4. Discussion

The findings of the present study reveal that most individuals adapt their decision-making strategies by prioritizing extensive evidence gathering when faced with uncertainty (Mushtaq et al., 2011). However, a subset of participants exhibited a tendency to jump to conclusions (JTC), as identified by the high and consistent correlations between the number of boxes opened across conditions in the JTC task (>0.70). Remarkably, this tendency persisted even under conditions designed to minimize the likelihood of such bias, suggesting that evidence-gathering strategies are relatively stable within individuals. This stability underscores the importance of identifying individual decision-making profiles, particularly in contexts where deficits under uncertainty might be detectable and modifiable. Such findings align with existing literature emphasizing the role of individual factors in understanding and addressing decision-making deficits (Brand et al., 2006; Gratton et al., 2018).

The second significant insight from this study is the role of cognitive control processes in decision-making biases. When comparing the three clusters, both cognitive stability (measured via the Flanker–Simon Task) and cognitive flexibility (measured via the Letter–Number Task) effectively differentiated individuals with a high tendency toward JTC (Cluster 1) from those in the other two clusters. Individuals with lower scores in both stability and flexibility are less able to maintain focus on task-relevant information or adapt their strategies in response to changing conditions. This finding aligns with prior research highlighting the role of executive functioning, particularly its shifting and updating components, in risk perception and judgment consistency (Missier et al., 2012). These cognitive deficits may partially explain the predisposition to JTC under uncertainty. Additionally, the observed independence between cognitive stability and flexibility, as indicated by negligible correlations between the Flanker and Letter–Number tasks, supports theoretical models treating these constructs as distinct rather than opposing dimensions (Dreisbach & Fröber, 2019; Egner & Siqi-Liu, 2024).

Decision-making in real-world environments is inherently multidimensional, driven by both appetitive and aversive motives (Wurm & Steinhauser, 2022). Gray’s Behavioral Approach System (BAS) and Behavioral Inhibition System (BIS) provide a useful framework for understanding individual differences in decision-making tendencies (Aluja et al., 2019). BIS is linked to heightened sensitivity to negative stimuli, increasing the perception of punishment (Lerner et al., 2024) and leading to anxiety, stress, and behavioral disorders such as obsessive–compulsive disorder or borderline personality disorder (Caprara & Cervone, 2000; Gangemi et al., 2021; Mancini et al., 2004; Masland & Hooley, 2020). Conversely, BAS dysregulation has been implicated in impulsive tendencies and pathological conditions, influencing decision-making patterns that favor immediate over delayed rewards (Kim & Lee, 2011; Sonuga-Barke et al., 2016; Urošević et al., 2008). While these theoretical models highlight the role of emotional–motivational systems in shaping decision-making, our study did not find a significant association between JTC and BIS/BAS measures. One possible explanation is that, in accordance with Cattell’s guidelines on objective testing (Cattell & Warburton, 1967), the Box Task omits explicit reward or punishment elements and withholds feedback to ensure that decision-making processes reflect intrinsic behavioral tendencies rather than being influenced by external cues.

Nevertheless, previous studies suggest that incorporating emotional dimensions, such as reward or punishment manipulations, could better capture the role of motivational systems in decision-making (Kim & Lee, 2011). Emotions influence decision-making in a context-dependent manner, shaping risk propensity and evaluative processes (Franco & Sanches, 2016), while also reinforcing pre-existing biases and belief structures (Lerner et al., 2015). Affective responses can distort judgment, with risk perception and decision strategies being guided more by emotional states than objective information (Ferrer et al., 2015; Han et al., 2007). These findings emphasize the need to further investigate how cognitive control and emotional factors interact, particularly in uncertain and high-risk environments.

Theoretical implications of the present findings include support for models that treat cognitive stability and flexibility as independent constructs rather than as two ends of a single continuum. Although some studies suggest an inverse relationship between stability and flexibility (Dreisbach & Fröber, 2019), our findings—characterized by negligible correlations between the Flanker–Simon and Letter–Number tasks—align with models proposing distinct mechanisms for maintenance (task focus) and updating (task switching) functions (Geddert & Egner, 2022). This independence opens avenues for interventions targeting one cognitive control process without adversely affecting the other. Additionally, emerging evidence suggests that stability and flexibility are linked to separate neural mechanisms, further supporting their distinct roles in adaptive decision-making (Egner & Siqi-Liu, 2024).

Practical recommendations based on this study’s findings include the importance of individualized profiling in interventions. While most individuals align their decision-making strategies with task demands, a subset consistently exhibits biased behavior. These individuals are at greater risk for psychiatric and neurological disorders, such as pathological gambling, eating disorders, and risky behaviors like unsafe driving (Catalan et al., 2022; Krężołek et al., 2019; Sanchez & Dunning, 2021). Tailored interventions focusing on these high-risk groups could mitigate their predisposition to maladaptive decision-making, ultimately improving outcomes in health, safety, and economic welfare.

Interventions should also target cognitive stability and flexibility processes to suppress decision-making deficits and biases. Evidence suggests that cognitive training can enhance flexibility and, in some cases, lead to generalized improvements across other cognitive and behavioral domains (Johann & Karbach, 2020). For example, interventions that emphasize executive functioning and calculative strategies over intuitive approaches have shown promise in reducing biases and enhancing decision-making under risk (Uddin, 2021). Moreover, individuals with higher cognitive abilities—often associated with superior executive functioning and working memory—tend to make more advantageous decisions, even in challenging conditions (Brand et al., 2009).

### Limitations and Future Directions

The present study has several limitations that also provide important avenues for future research. A key limitation is the use of a convenience sample consisting primarily of first-year undergraduate students in psychology, which introduces potential biases related to age, educational background, and cognitive development. Research suggests that student samples, while widely used for their accessibility, may not be fully representative of broader populations. For example, Hanel and Vione (2016) argue that personality and attitudinal differences between students and the general public can hinder the generalization of findings. Similarly, Novielli et al. (2023) found significant statistical discrepancies in marketing and consumer behavior research when relying on undergraduate students, while Peterson and Merunka (2014) highlighted how traditional student-based sampling methods may compromise external validity. These findings underscore the importance of incorporating more diverse and representative samples in future studies to enhance generalizability and ensure the robustness of theoretical models.

Further research is needed to explore the interaction between personality traits, particularly BIS/BAS tendencies, and decision-making biases in uncertain contexts. Although no significant association was found in this study, the observed divergence between self-report measures and objective performance tasks suggests that dispositional personality traits may not always directly translate into behavioral decision-making patterns. This discrepancy, rather than being dismissed as a methodological issue, highlights the complexity of integrating self-reported personality constructs with cognitive control processes assessed through behavioral tasks. Future research should investigate the mechanisms underlying this divergence to refine models of personality-influenced decision-making and assess whether specific personality profiles, such as those characterized by heightened anxiety or impulsivity, contribute to biased decision-making under uncertainty. Identifying these profiles could provide a basis for developing tailored psychological interventions aimed at improving decision-making strategies.

Additionally, the ecological validity of experimental paradigms such as the JTC Box Task warrants further refinement. While these tasks are not designed to replicate real-life decision-making scenarios, they aim to model the functional relationships that drive decision-making in controlled settings. Enhancing these paradigms to better reflect these functional relationships—without oversimplifying the complexity of real-world decisions—would strengthen their applicability and relevance. One promising direction may involve the incorporation of reward or punishment manipulations into decision-making tasks to better capture the influence of motivational systems (Kim & Lee, 2011). Such modifications could provide deeper insights into how external reinforcements modulate cognitive control and decision-making biases, further elucidating the role of BIS/BAS traits in these processes.

Moreover, this study found cognitive stability and flexibility to be independent constructs, suggesting the need for further research into their distinct neural mechanisms. Prior studies indicate that these processes involve separate regulatory pathways (Egner & Siqi-Liu, 2024; Geddert & Egner, 2022). Investigating these mechanisms in greater depth could refine our understanding of their roles in adaptive and maladaptive decision-making. Expanding the range of cognitive control assessments beyond those used in this study—such as incorporating measures of working memory, inhibitory control, and planning—would provide a more comprehensive picture of how executive functions contribute to decision-making under uncertainty.

Finally, future research should extend these investigations to clinical populations, where decision-making biases are often more pronounced. Examining how cognitive control mechanisms interact with psychiatric and neurological disorders could help identify potential pathways for targeted interventions aimed at mitigating maladaptive decision-making patterns. Addressing these areas will contribute to refining theoretical models and developing strategies to improve decision-making in both clinical and everyday contexts.

## 5. Conclusions

The present study underscores the importance of cognitive control mechanisms, particularly stability and flexibility, in shaping decision-making strategies under uncertainty. By demonstrating how these processes differentiate individuals prone to jumping to conclusions from those adopting more cautious approaches, the findings contribute to a nuanced understanding of decision-making biases. The independence observed between stability and flexibility further supports their treatment as distinct constructs, offering valuable insights for theoretical models and interventions aimed at mitigating maladaptive decision-making.

While the study highlights the utility of the Box Task in exploring evidence-gathering behaviors, it also emphasizes the need for more diverse and representative samples to generalize findings beyond student populations. Additionally, refining experimental tasks to better model real-world functional relationships remains a key priority for future research. Integrating emotional and motivational dimensions into decision-making paradigms could further elucidate the role of personality traits, such as BIS and BAS, in shaping decision-making tendencies.

Ultimately, the findings provide a foundation for developing tailored interventions targeting cognitive deficits associated with maladaptive decision-making. By addressing these mechanisms in high-risk groups, such interventions hold promise for improving outcomes across clinical, economic, and societal domains.

## Figures and Tables

**Table 1:** Descriptive statistics and correlation matrix for the variables included in the study.

	1	2	3	4	5	6	7	8	9
1. BT_1	-	0.755 **	0.696 **	0.009	0.062	−0.033	0.043	0.101	0.069
2. BT_2			0.860 **	−0.003	−0.001	−0.006	−0.016	0.172 *	0.122
3. BT_3				0.041	0.042	−0.017	−0.002	0.212 **	0.129
4. BIS					0.044	−0.020	0.004	0.039	−0.042
5. BAS						−0.085	−0.039	−0.110	0.066
6. CS-AC							0.081	0.130	−0.064
7. CS-Dif								0.012	0.164 *
8. CF-AC									0.013
9. CF-Dif									-
N	193	194	193	195	195	185	185	186	186
Mean	11.92	14.27	21.87	22.24	39.86	86.77	53.09	61.82	583.3151
SD	2.83	2.41	4.94	3.20	4.69	3.28	51.59	2.00	337.8239

Note: BT = Box Task, CS = Cognitive Stability, CF = Cognitive Flexibility; 1. BT Condition 1; 2. BT Condition 2; 3. BT Condition 3; 4. BIS score; 5. BAS score; 6. CS accuracy; 7. CS differences in RTs; 8. CF accuracy; 9. CF differences in RTs. * *p* < 0.05. ** *p* < 0.01.

**Table 2:** Descriptive statistics and ANOVA results for evidence-gathering behavior and cognitive measures across the three clusters.

	Cluster 1 Minimal Evidence Gatherers			Cluster 2 Moderate Evidence Gatherers			Cluster 3 Extensive Evidence Gatherers			*F*	*p*
	N	Mean	SD	N	Mean	SD	N	Mean	SD		
1. BT_1	24	7.81	1.388	41	9.27	1.438	118	13.58	1.672	73.526	0.000 **
2. BT_2	24	9.31	1.762	41	13.27	1.753	118	15.57	0.672	223.095	0.000 **
3. BT_3	24	10.93	2.425	41	20.16	3.638	118	24.52	0.877	1202.874	0.000 **
4. BIS	24	22.29	4.016	40	22.08	2.411	115	22.30	3.199	0.167	0.846
5. BAS	24	39.29	5.129	40	39.55	3.948	115	39.97	4.659	0.357	0.700
6. CS-AC	23	48.57	48.821	37	58.76	55.790	111	52.76	53.075	3.451	0.034 *
7. CS-Dif	23	87.39	3.056	37	86.35	3.698	111	86.71	3.326	0.168	0.846
8. CF-AC	22	61.05	2.340	39	61.69	2.353	112	62.04	1.823	5.533	0.005 **
9. CF-Dif	22	408.79	232.618	39	654.86	352.977	112	568.08	310.677	5.513	0.005 **

Note: BT = Box Task, CS = Cognitive Stability, CF = Cognitive Flexibility; 1. BT Condition 1; 2. BT Condition 2; 3. BT Condition 3; 4. BIS score; 5. BAS score; 6. CS accuracy; 7. CS differences in RTs; 8. CF accuracy; 9. CF differences in RTs. * *p* < 0.05. ** *p* < 0.01.

**Table 3:** Multinomial logistic regression of cognitive control measures as predictors of cluster membership.

				95% CI		
	*B*	SE	OR	Lower	Upper	*p*
**Cluster 2 (Moderate) vs. 1 (Minimal)**						
Flanker–Simon Task (Correct)	−0.240	0.118	0.787	0.624	0.992	0.042
Letter–Number Task (Correct)	0.007	0.142	1.007	0.763	1.330	0.961
Letter–Number Task (Latency)	0.004	0.001	1.004	1.001	1.006	0.006
**Cluster 3 (Extensive) vs. 1 (Minimal)**						
Flanker–Simon Task (Correct)	−0.159	0.106	0.853	0.694	1.050	0.134
Letter–Number Task (Correct)	0.264	0.118	1.302	1.033	1.641	0.026
Letter–Number Task (Latency)	0.003	0.001	1.003	1.001	1.005	0.006
**Cluster 3 (Extensive) vs. 2 (Moderate)**						
Flanker–Simon Task (Correct)	−0.081	0.065	0.922	0.812	1.047	0.212
Letter–Number Task (Correct)	−0.257	0.108	0.774	0.626	0.956	0.017
Letter–Number Task (Latency)	0.000	0.001	1.000	0.999	1.002	0.570

Note: OR = Odds Ratio, CI = Confidence Interval.

## Data Availability

The datasets presented in this article are not readily available because they form part of an ongoing broader project and may be included in future publications. Requests to access the datasets should be directed to pei_chun.shih@uam.es.
